# Sex-dependent treatment of chronic EAE with partial MHC class II constructs

**DOI:** 10.1186/s12974-017-0873-y

**Published:** 2017-05-06

**Authors:** Gil Benedek, Priya Chaudhary, Roberto Meza-Romero, Evan Calkins, Gail Kent, Halina Offner, Dennis Bourdette, Arthur A. Vandenbark

**Affiliations:** 1 Neuroimmunology Research, VA Portland Health Care System, Portland, OR USA; 2 Tykeson MS Research Laboratory, Department of Neurology, Oregon Health & Science University, Portland, OR USA; 3 Department of Neurology, Oregon Health & Science University, Portland, OR USA; 4 Department of Anesthesiology and Perioperative Medicine, Oregon Health & Science University, Portland, OR USA; 5 Neurology Service, VA Portland Health Care System, Portland, OR USA; 6 Department of Molecular Microbiology & Immunology, Oregon Health & Science University, Portland, OR USA; 7 Research Service R&D31, VA Portland Health Care System, 3710 SW US Veterans Hospital Rd, Portland, OR 97239 USA

**Keywords:** Chronic experimental autoimmune encephalomyelitis (EAE), Multiple sclerosis (MS), RTL342M, DRα1-mMOG-35-55, Gender effect

## Abstract

**Background:**

One of the main challenges in treating multiple sclerosis (MS) is reversing the effects of accumulated damage in the central nervous system (CNS) of progressive MS subjects. While most of the available drugs for MS subjects are anti-inflammatory and thus are limited to relapsing-remitting MS subjects, it is not clear to what extent their effects are capable of inducing axonal repair and remyelination in subjects with chronic MS.

**Methods:**

A chronic model of experimental autoimmune encephalomyelitis (EAE) was used to evaluate the potency of partial MHC (pMHC) class II constructs in treating progressive EAE.

**Results:**

We demonstrated an estrogen receptor alpha (ERα)-dependent increased dose requirement for effective treatment of female vs. male mice using pMHC. Such treatment using 100-μg doses of RTL342M or DRα1-mMOG-35-55 constructs significantly reversed clinical severity and showed a clear trend for inhibiting ongoing CNS damage, demyelination, and infiltration of inflammatory cells into the CNS in male mice. In contrast, WT female mice required larger 1-mg doses for effective treatment, although lower 100-μg doses were effective in ovariectomized or ERα-deficient mice with EAE.

**Conclusions:**

These findings will assist in the design of future clinical trials using pMHC for treatment of progressive MS.

**Electronic supplementary material:**

The online version of this article (doi:10.1186/s12974-017-0873-y) contains supplementary material, which is available to authorized users.

## Background

Multiple sclerosis (MS) is a chronic, immune-mediated demyelinating disease of the central nervous system (CNS) [1–4]. MS is categorized into subtypes according to its clinical course: relapsing-remitting (RR) MS is dominated by episodic focal infiltration of the CNS by lymphocytes and monocytes, edema, and the physiologic actions of cytokines [5]. Progressive forms of MS are characterized by axonal degeneration in the absence of overt extrinsic acute inflammatory lesions [6–8], although episodic focal inflammatory lesions still occur, but at a much lower frequency than in RRMS. The pathogenesis of the progressive neuronal and axonal loss, which occurs throughout the CNS, in MS is uncertain, but the toxic effects of reactive oxygen species and other soluble mediators of inflammation released by activated microglia may be critical to this stage of the disease [4, 6, 9, 10].

Although significant progress has been made in understanding disease mechanisms in RRMS, our knowledge of the processes that lead to disease progression is limited. Furthermore, while there are several Food and Drug Administration (FDA)-approved therapies for the treatment of RRMS, the only approved drug for secondary progressive (SP) MS is a chemotherapy drug, mitoxantrone, which has limited benefit and is highly toxic [11]. Most of the immunotherapeutic drugs for RRMS have limited efficacy in treating progressive patients. Hence, it remains a formidable challenge to develop new therapies for MS that not only inhibit CNS inflammation but also promote remyelination and prevent or reduce axonal damage.

It was previously shown that partial MHC class II constructs (pMHC, also known as recombinant T cell receptor ligands (RTLs)), containing the extracellular domains of class II major histocompatibility complex (MHC) molecules linked covalently to specific peptides, could successfully treat established experimental autoimmune encephalomyelitis (EAE)—the murine model of MS. We also demonstrated that SJL/J mice with established EAE could be treated successfully with RTL401, a mouse I-A^s^/PLP-139–151 construct, after the peak of EAE. RTL-treated mice had reduced pathology compared with mice treated with vehicle and mice at the peak of disease, as demonstrated by a decrease in continued degeneration, an increase in remyelinated axons, and the presence of an increased number of small, presumably regenerative axonal sprouts [12].

Additional studies further indicated that these constructs could bind to and downregulate the expression of cell surface CD74 on antigen-presenting cells (APC) and competitively block the binding of macrophage migration inhibitory factor (MIF) and its downstream signaling [12–14]. We also discovered that the DRα1 domain, but not the DR2β1 domain of partial MHC class II constructs, binds to cell surface CD74 on human and mouse monocytes and that this interaction competitively blocks MIF binding to the CD74 trimerization domain [14, 15]. This interaction may be potentially exploited as a treatment for MIF/CD74-dependent CNS diseases such as progressive MS [15, 16]. We reported that the DRα1 domain (without a covalently linked peptide) could effectively treat EAE, but its potency could be enhanced ~50-fold by the addition of a peptide extension [15]. We further reported that an optimized DRα1-mouse (m)MOG-35-55 construct could effectively treat EAE in DR*1501-Tg and C6 mice at a clinical/6 WT mice, enhance the frequency of anti-inflammatory macrophages and microglial cells in the spinal cord, and promote the expression of neuroprotective genes [17].

Herein, we demonstrate sex-dependent effects of partial MHC class II constructs to reverse EAE clinical signs and partially arrest demyelination and neuronal damage in the CNS.

## Methods

### Mice

C6 mice at a clinical/6 WT males and females, ovariectomized females, and estrogen receptor (ER)α and ERβ knockout (KO) females were purchased from the Jackson Laboratory. DR*1501-Tg mice were bred in-house at the Veterinary Medical Unit, VA Portland Health Care System, and used at 8–12 weeks of age. In order to allow complete recovery form operation, ovariectomized females were housed for 2 weeks before starting any procedure. All procedures were approved and performed according to federal, state, and institutional guidelines.

### Partial MHC class II design and production

The design, production, and characterization of the partial MHC class II constructs have been described elsewhere [15, 18]. In the DR*1501-Tg mice experiments, RTL342M, which contains the human HLA-DRB1*1501 β1α1 domains covalently linked to a murine MOG-35-55 peptide, was used. In the other experiments, DRα1-mMOG-35-55, which contains the human HLA-DRa1 domain covalently linked to a mouse MOG-35-55 peptide, was used. DRα1-MOG was built using the α1 domain construct as a template. The mouse MOG-35-55 peptide DNA encoding sequence was attached to the N-terminus of the DRα1 domain with a linker-thrombin-linker intervening element. The single-chain gene was cloned into the *Nco*I and *Xho*I restriction sites of the pET21d(+) vector (Novagen) and transformed into *E. coli* BL21 (DE3) expression host (Stratagene). For protein production, 4 L of Luria-Bertani medium, supplemented with 50 μg/ml of carbenicillin, was inoculated with a starting OD_600_ of 0.05. IPTG was added when the culture reached 0.7 OD_600_. Cultures were allowed to grow for 4 h and then ice-chilled before harvesting at 7000 rpm for 6 min. After centrifugation, the pellet was resuspended in lysis buffer (50 mM Tris, 5 mM EDTA, 300 mM NaCl, pH 8), treated with lysozyme (1 ml at 10 mg/ml) for 30 min at room temperature, and lysed in ice by sonication in a Branson Sonifier 450 apparatus with pulses of 1 min and pauses of 5 min. The disrupted suspension was pelleted at 7000 rpm for 6 min, and the paste was resuspended in 1% Triton X-100 in lysis buffer to remove lipids and other hydrophobic contaminants. Detergent was removed by resuspending the pellet in lysis buffer followed by sonication as described earlier. This was repeated three more times. The final pellet was solubilized in buffer A (20 mM ethanolamine, 6 M urea, pH 10) overnight at 4 °C and then spun down at 40,000*g* to remove particulate material. This lysate was filtered through a 0.22-μm filter twice, loaded onto a Mono Q anion-exchange 50-ml column at a flow rate of 2 ml/min attached to an AKTA FPLC (GE Healthcare). After washing the column until no eluting material was detected at 280 nm, proteins were eluted by applying a stepwise gradient of 2 M NaCl in buffer A. The eluate was collected in fractions of 8 ml ,and after electrophoretic analysis, those containing the target protein were pooled together. This pooled material was concentrated with a 3-kDa MWCO membrane (Millipore), filtered twice through a 0.22-μm membrane and then loaded onto a Superdex 75, 16/60 size exclusion column (GE Healthcare). The loaded protein was eluted with buffer C (20 mM ethanolamine, 4 mM NaCl, 6 M urea, pH 10) at a flow rate of 1 ml/min, collected into fractions of 1 ml and finally dialyzed against 20 mM Tris, pH 8.5, for refolding. After refolding, the DRα1-MOG protein was concentrated to 10 mg/ml, snap-frozen, and stored in 1-ml aliquots at −80 °C until use.

### Induction of EAE

DR*1501-Tg mice were screened for the expression of the HLA marker by flow cytometry [19]. Male and female mice between 8 and 12 weeks of age were immunized subcutaneously (s.c.) at four sites on the flanks with 0.2 ml of an emulsion of 200 μg immunogenic peptide and complete Freund’s adjuvant (CFA) containing 400 μg (DR*1501-Tg mice) or 200 μg (C6 mice at a clinical/6 or estrogen receptor-deficient mice) of heat-killed *Mycobacterium tuberculosis* H37RA [19] (Difco, Detroit, MI). DR*1501-Tg mice require a higher concentration of CFA in order to present equivalent disease severity as C6 mice at a clinical/6 mice immunized with a lower concentration of CFA. In addition, mice were given pertussis toxin (Ptx) from List Biological Laboratories (Campbell, CA) on days 0 and 2 post-immunization (75 and 200 ng per mouse, respectively). Immunized mice were scored blinded to treatment status for clinical signs of EAE graded on a 6-point scale of combined hind limb and forelimb paralysis scores. For hind limb scores, 0 = no signs; 0.5 = limp tail or mild hind limb weakness (i.e., a mouse cannot resist inversion after a 90° turn of the base of the tail); 1 = limp tail and mild hind limb weakness; 2 = limp tail and moderate hind limb weakness (i.e., an inability of the mouse to rapidly right itself after inversion); 3 = limp tail and moderately severe hind limb weakness (i.e., inability of the mouse to right itself after inversion and clear tilting of hind quarters to either side while walking); 4 = limp tail and severe hind limb weakness (hind feet can move but drag more frequently than face forward); 5 = limp tail and paraplegia (no movement of hind limbs). Front limb paralysis scores are either 0.5 for clear restriction in normal movement or 1 for complete forelimb paralysis. The combined score is the sum of the hind limb score and the forelimb score. Rarely, there is mortality of mice with severe EAE and in these cases; mice are scored as a 6 for the remainder of the experiment. Mean EAE scores and standard deviations for mice grouped according to initiation of RTL342M, DRα1-mMOG-35-55, or vehicle treatment were calculated for each day and summed for the entire experiment (cumulative disease index (CDI) represents total disease load).

### Partial MHC class II treatment of chronic EAE

To treat EAE induced in C57BL/6 and DR*1501-Tg mice, 100 μg or 1 mg RTL342M or DRα1-mMOG-35-55 protein was injected s.c. daily for 5 days beginning on day 20 post-disease induction. Additionally, mice received boosting treatments of three daily s.c. injections on days 35 and 49 p.i. Clinical signs were scored as described above.

### Quantitative morphological determination of percentage of tissue damage and demyelination

At the end of the experimental treatment, mice were euthanatized and the spinal columns were isolated and fixed overnight in 4% paraformaldehyde. After 24 h, the spinal cords were isolated and microwaved for 1.15 h using the BioWave Pro. The lumbar spinal cord was used for quantifying inflammation by immunohistochemistry using anti-CD4 and anti-CD11b antibodies and histology. For histology, the spinal cord was embedded in paraffin and plastic. Myelination assessment was done using luxol fast blue-periodic acid-Schiff (LFB-PAS) staining at the light level. Axon integrity was determined by toluidine blue staining in plastic-embedded spinal cord sections using light microscopy. All measurements were performed blinded to treatment status.

For plastic embedding, the spinal cord was further fixed in 1.5% paraformaldehyde and 1.5% glutaraldehyde solutions. Tissue samples were treated with 1% osmium, dehydrated, and embedded in resin using BioWave Pro. Semi-thin sections (0.5 μm) were stained with toluidine blue and photographed using a Zeiss Axio Imager 2 bright-field microscope. Photomontages of the entire spinal cord cross sections were made: areas containing damaged axons were manually marked at ×20 in MetaMorph software, v7.7.5 (Molecular Devices, CA). The ventro-lateral white matter area was determined by outlining the ventro-lateral spinal cord. Degenerating axons with abnormal morphology, myelin ovoids, unstable myelin, and areas with absence of axons were marked as damaged. The damaged areas were added together, and the percentage of damaged area was determined for each spinal using the following formula: area of damage/total area × 100%. In addition, axon counts were determined from eight images (×63) from the ventro-lateral area in each mouse. A 50 μm × 50 μm square was analyzed in each image.

To assess the effects of RTL342M and DRα1-MOG-35-55 on demyelination, spinal cord sections were stained with LFB-PAS and the area of demyelination determined. For myelin staining and analysis, part of the lumbar spinal cord was embedded in paraffin, sectioned, and stained with LFB-PAS. Briefly, the slides were deparaffinized and brought to 95% ethanol. Staining in luxol fast blue solution (Sigma-Aldrich, St. Louis, MO) was done overnight at 60 °C. The slides were rinsed with deionized water and placed in 0.5% periodic acid for 10 min. After rinsing with deionized water, the slides were placed in Schiff’s reagent (Sigma-Aldrich, St. Louis, MO) for 10 min. The slides were washed in running tap water for 10 min and nuclei-stained with Harris hematoxylin (Thermo Fisher Scientific, NJ) for 1 min. After washing the slides in tap water, the slides were dipped in saturated lithium carbonate to remove excess stains (5–10 dips). The slides were rinsed again and dehydrated with different grades of alcohol. The slides were cleared in xylene and mounted with synthetic mounting media. The percentage of spinal cord demyelination was calculated by determining the total area of white matter by manually tracing the regions in MetaMorph (version 7.7.5, Molecular Devices, CA) and marking the area of demyelination in the ventro-lateral spinal cord. The percentage of demyelination was obtained by dividing the area of demyelination by the total area.

### Quantitative immunohistochemical studies

For quantitative immunofluorescent analyses of the spinal cord, 50-μm sections were randomly selected for antibody staining. Briefly, the sections were permeabilized, washed, blocked (0.5% fish skin gelatin/3% BSA) in PBS, and then incubated with primary antibodies at 4 °C overnight. Anti-CD4 (BD Pharmingen, San Diego, CA; 1:25 dilution) and anti-Mac-1 (CD11b; Leinco Technologies, St. Louis, MO; 1:75 dilution) were used to identify T cells and microglia/macrophage populations, respectively. After washing, the sections were incubated in secondary antibody, Alexa Fluor 488 donkey anti-rat IgG (Invitrogen, Carlsbad, CA; A21208; 1:200 dilution). The sections were then washed and mounted in Prolong Gold antifade. To quantify immunofluorescence, the sections were imaged (×40 objective, images were taken from dorsal, ventral, left lateral and right lateral spinal cord) on a Zeiss confocal microscope (LSM 780). Twelve-bit images were captured at a resolution of 1024 × 1024 pixels using an UplanF1 ×40/1.3 oil-immersion objective with Zen software. Data analyses were performed using MetaMorph software v7.7.5 (Molecular Devices, CA). Negative controls were generated by omitting the primary antibody. An additional control comprised omission of both primary and secondary antibodies. Thresholding and intensity measurement methods of image analysis were used. Similar methods have been used for quantification of CD4^+^ and CD11b^+^ cells in the spinal cords of mice with EAE [20–22].

### Statistical analysis

Daily mean scores were analyzed statistically by a two-tailed Mann-Whitney *U* test for a nonparametric comparison between vehicle and RTL342M or DRα1-mMOG-35-55 treatment groups. Mean CDIs were analyzed by Student’s *t* test. Values of *p* < 0.05 were considered significant.

## Results

### RTL342M treats chronic EAE and reduces cellular infiltration and CNS damage in male DR*1501-Tg mice

**Fig. 1:**
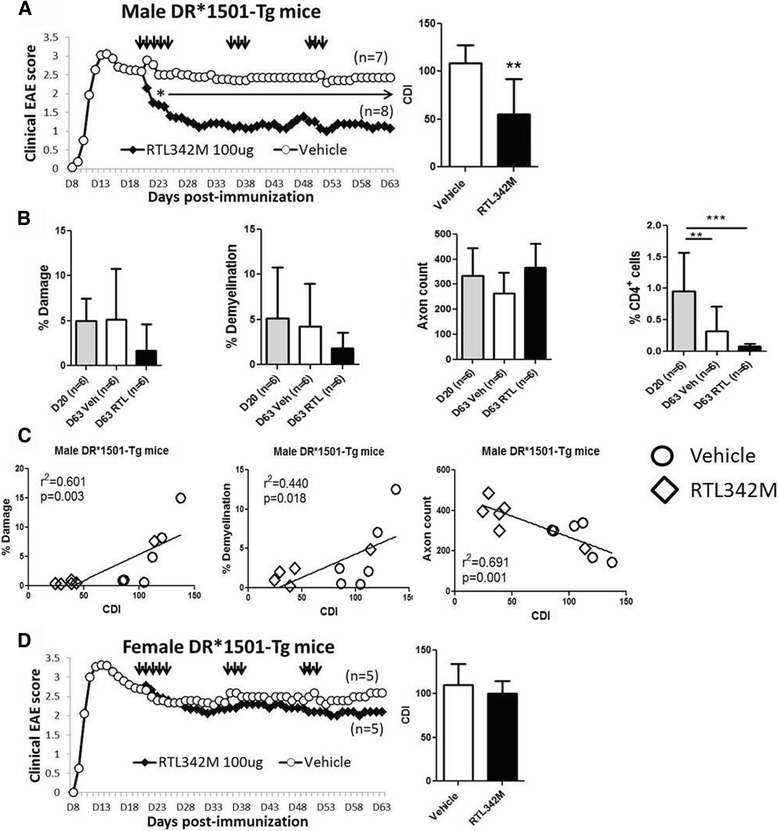
RTL342M improves clinical scores and reduces neurohistological damage in male DR*1501-Tg mice with chronic EAE. **a** Mean EAE daily scores of male DR*1501-Tg mice (treatment days indicated by *black arrows*) (*left*) and cumulative disease indices (*right*). *p ≤ 0.05, **p ≤ 0.01. Daily mean scores were analyzed by Mann-Whitney *U* and mean CDI by Student’s *t* test. **b** Days 20 and 63 p.i. spinal cord lumbar sections from EAE DR*1501-Tg mice were stained with luxol fast blue (LFB) and analyzed for demyelination and with toluidine blue for spinal cord damage and axon counts. *Far right*: CD4^+^ T cell frequencies were assessed in the ventro-lateral area of spinal cords of RTL342M- vs. vehicle-treated male mice. **p<0.01, ***p<0.001, one way ANOVA. **c** Correlation plots of % damage, % demyelination, and axon counts vs. CDI for male mice on day 63 post-immunization. **d** Mean EAE daily scores of female DR*1501-Tg mice (treatment days indicated by *black arrows*) (*left*) and cumulative disease indices (*right*)

While prophylactic treatment of EAE can be achieved with various agents, including pMHC constructs, treatment of active disease at onset or especially at disease peak, when demyelination and axonal damage are already established, is more challenging. Previously, we demonstrated successful treatment of SJL/J mice with chronic relapsing EAE using RTL401, a mouse I-A^s^/PLP-139–151 construct [12]. Here, we further evaluate the ability of a different RTL construct to reverse disease progression in a chronic EAE model induced with MOG-35-55 peptide on a humanized HLA-DR2 genetic background. Thus, 21 male and 16 female DR*1501-Tg mice were immunized with mMOG-35-55/CFA/Ptx. Mice were treated with vehicle or RTL342M (a partial DR2 MHC class II single-exon construct comprised of the β1α1 domains of DR2 tethered to mMOG-35-55 peptide) commencing on day 20 (100 μg daily × 5), with booster injections on days 35 and 49 (100 μg daily × 3) as indicated by black arrows in Fig. 1a for male mice (RTL342M *n* = 8, vehicle *n* = 7) and in Fig. 1d for female mice (RTL342M *n* = 5, vehicle *n* = 5). Before treatment, on day 20 p.i. or on day 63 p.i., the spinal cords were analyzed for infiltrating cells, demyelination by LFB-PAS staining, and tissue damage (number of axons) by toluidine blue staining of the lumbar section of the spinal cords (Fig. 1b).

The cumulative disease index scores (CDI = total EAE clinical disease load from onset of treatment until the end of the experiment on day 63) did not differ between vehicle-treated males and females (Fig. 1a, d), indicating comparable disease severity. A comparison of disease course between male and female DR*1501-Tg mice is presented in Additional file 1: Figure S1A. However, male mice treated with RTL342M had early reductions and significantly lower CDIs on day 63 compared with vehicle-treated male mice (Fig. 1a, 55.3 ± 36 vs. 108.3 ± 18, respectively, *p* < 0.01) as well as a possible trend in inhibiting further tissue damage (% damage: day 20 = 4.9 ± 2.5, day 63 vehicle = 5.1 ± 5.6, and day 63 RTL342M = 1.7 ± 2.9) and ongoing demyelination (% demyelination: day 20 = 4.3 ± 3.6, day 63 vehicle = 4.1 ± 4.7, and day 63 RTL342M = 1.8 ± 1.7) (Fig. 1b). Nominally reduced axon counts in vehicle-treated male mice also trended higher to day 20 levels in the RTL342M-treated male mice by day 63 (axon count: day 20 = 333 ± 110, day 63 vehicle = 263 ± 84, and day 63 RTL342M = 365 ± 95, *p* < 0.07). The spinal cords from two RTL342M- and one vehicle-treated mice were used for different assay (not reported) and were not included in this analysis. Further analysis of these trends of the vehicle- and RTL342M-treated mice on day 63 demonstrated highly significant correlations between the clinical vs. histological data, with more damage (*p* < 0.003), demyelination (*p* < 0.01), and lower axon counts (*p* < 0.001) with increased CDIs (Fig. 1c), lending histological support to the improved clinical outcome observed in five of six RTL342M-treated mice. Unexpectedly, female mice treated with the same 100-μg dosing regimen with RTL342M as males did not differ significantly in CDI from vehicle-treated female mice on day 63 (Fig. 1d), nor were there any trends suggesting reduced CNS damage, demyelination, or infiltrating CD4^+^ T cells (Additional file 1: Figure S2). Since no treatment effect was observed, axon counts were not performed for female mice.

### Sex-dependent efficacy of DRα1-mMOG-35-55 in treating chronic EAE

**Fig. 2:**
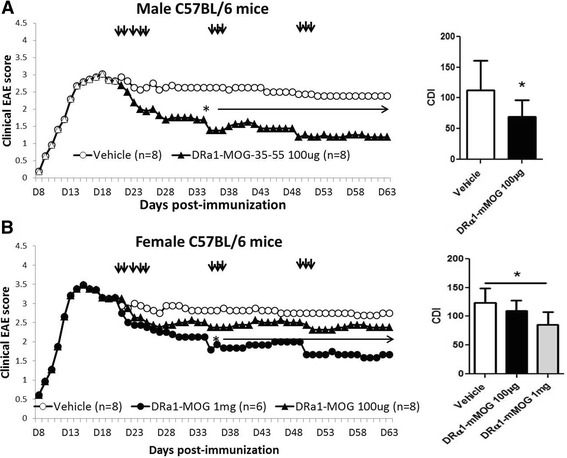
DRα1-mMOG-35-55 treatment of female C57BL/6 mice with chronic EAE requires increased dose vs. males. Mean daily scores of **a** male and **b** female C57BL/6 mice with chronic EAE treated with 1-mg or 100-μg doses of DRα1-mMOG-35-55 or vehicle (injections indicated by *black arrows*) (*left*) and cumulative disease indices (*right*). *p ≤ 0.05. Daily mean scores were analyzed by Mann-Whitney *U* and mean CDI by Student’s *t* test

**Fig. 3:**
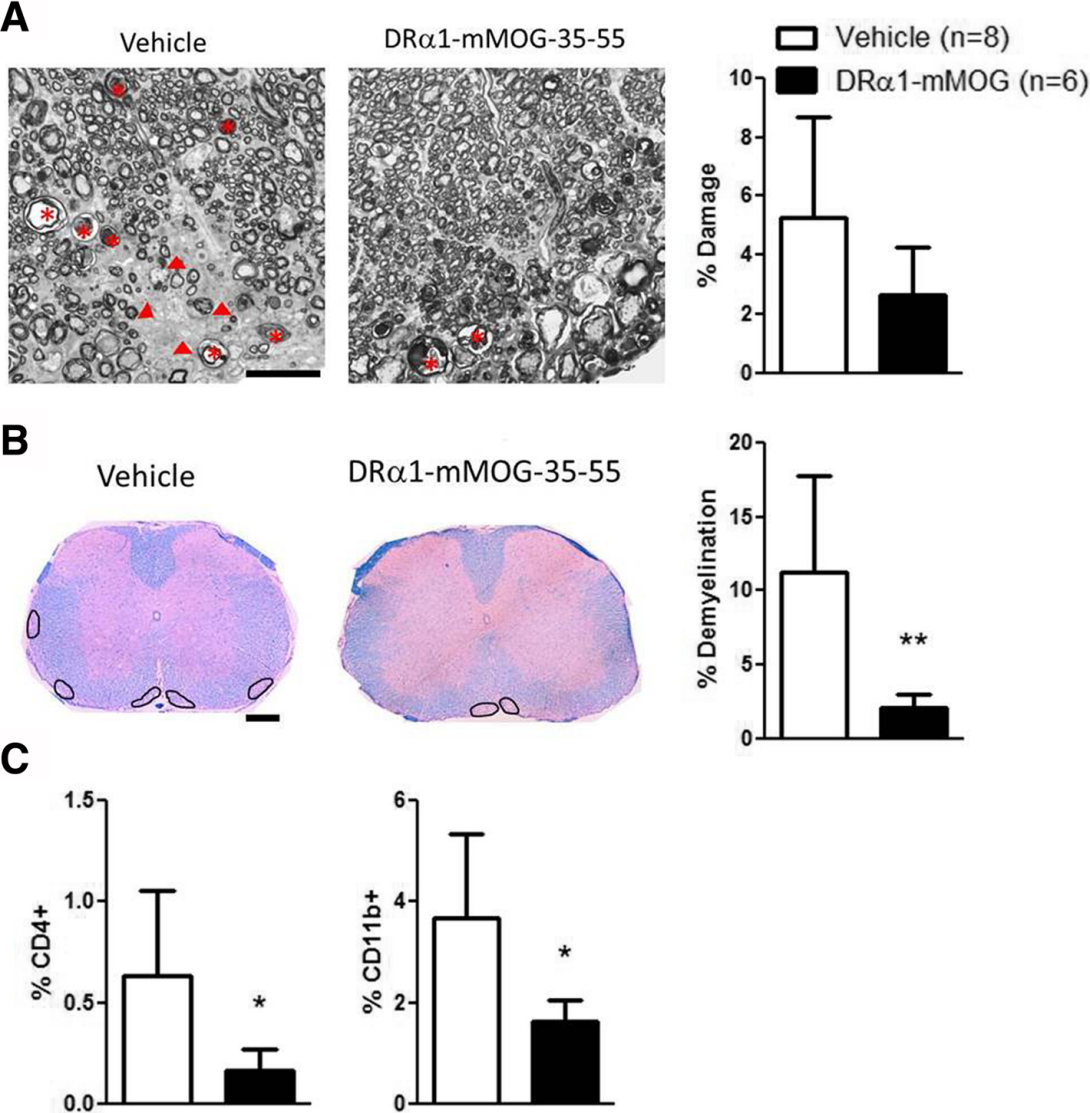
High dose of DRα1-mMOG-35-55 reduces demyelination and leukocyte infiltration in female mice with chronic EAE. **a** High-magnification (×63) plastic-embedded spinal cord cross sections are shown from mice 63 days post-immunization after staining with toluidine blue lateral spinal cord of vehicle- or 1 mg DRα1-mMOG-35-55-treated female C57BL/6 mice. *Arrow heads* show lesions. *Asterisks* show degenerating axons. *Scale bar* is 20 μm. Percentage of damage is shown in the bar graph (*right*) **b** Paraffin-embedded sections (×20) were used for assessing demyelination using LFB-PAS and hematoxylin staining at 63 days post-immunization. A few areas of demyelination are marked on the sections from DRα1-mMOG-35-55- or vehicle-treated mice. *Scale bar* is 200 μm. Percentage of demyelination is shown in bar graph (*right*). **p ≤ 0.01. Student’s *t* test. **c** Frequencies of CD4^+^ and CD11b^+^ cells in spinal cord lumbar section of vehicle- or 1 mg DRα1-mMOG-35-55-treated female C57BL/6 mice as evaluated by immunofluorescent staining. *p ≤ 0.05. Student’s *t* test

Previously, we demonstrated that our second-generation pMHC construct, DRα1-mMOG-35-55, could successfully treat EAE when injected into either HLA-“matched” DR2-Tg mice or HLA-“mismatched” C6 mice at a clinical/6 mice at a clinical score of ≥2 [15, 17]. We thus tested the ability of DRα1-mMOG-35-55 to treat chronic EAE in mismatched C57BL/6 mice and found the same sex-dependent pattern of treatment, with the 100-μg dose producing a significant improvement in chronic EAE in males (*p* < 0.05) but not females (Fig. 2). These data were reminiscent of the same sex-dependent response to treatment in experimental stroke, but in that case, females unresponsive to the 100-μg dose could be effectively treated with a 10-fold higher 1000-μg dose of DRα1-mMOG-35-55 [23]. As demonstrated for the DR*1501-Tg mice, there were no statistical differences in the disease course or the CDI between vehicle-treated male and female C57BL/6 mice (Additional file 1: Figure S1B). Thus, we repeated the experiment in female C57BL/6 mice with chronic EAE using a 1-mg dose of DRα1-mMOG-35-55 and found a marked early and significant cumulative treatment effect similar to the low-dose treatments in male mice (*p* < 0.05; Fig. 2, bottom). Analysis of the spinal cords from DRα1-mMOG-35-55-treated female mice revealed nominally less axonal damage compared to vehicle-treated mice (2.6 ± 0.6 vs. 5.2 ± 1.2%, respectively, Fig. 3a), significantly less demyelination (2.1 ± 0.3 vs. 11.2 ± 2.3%, respectively, *p* < 0.01, Fig. 3b), and reduced inflammation marked by significantly reduced frequencies of CD11b^+^ (1.6 ± 0.1 vs. 3.6 ± 0.5%, respectively, *p* < 0.05) and CD4^+^ T cells (0.16 ± 0.04 vs. 0.6 ± 0.14%, respectively, *p* < 0.05) (Fig. 3c). These results suggested that the difference in effective dose for treatment of chronic EAE with DRα1-mMOG-35-55 is sex dependent

### DRα1-mMOG-35-55 treatment efficacy of female mice depends on estrogen signaling through ERα

**Fig. 4:**
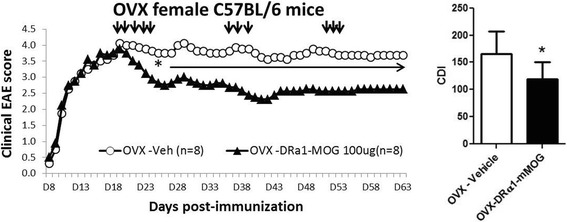
DRα1-mMOG-35-55 treatment efficacy of chronic EAE in female mice depends on sex hormones. Mean EAE daily scores of OVX female C6 mice at a clinical/6 mice treated with 100 μg DRα1-mMOG-35-55 or vehicle (injection days indicated by *black arrows*) (*left*) and cumulative disease index scores (*right*) are shown. *p ≤ 0.05. Daily mean scores were analyzed by Mann-Whitney *U* and mean CDI by Student’s *t* test

**Fig. 5:**
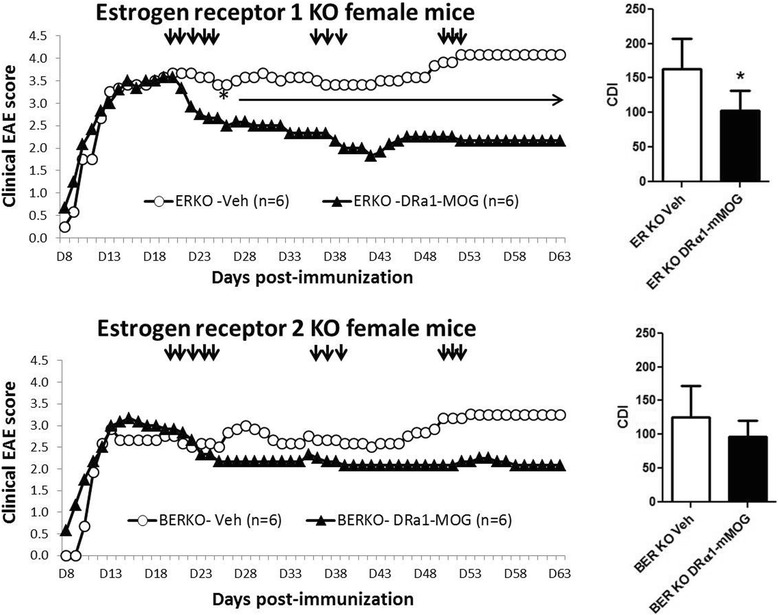
DRα1-mMOG-35-55 treatment efficacy of chronic EAE in female mice depends on the expression of ERα. EAE daily scores of ERα-KO (*ERKO*) and ERβ-KO (*BERKO*) female mice treated with 100 μg DRα1-mMOG-35-55 or vehicle (injection days indicated by *black arrows*) and mean clinical EAE daily disease scores (*left*) and cumulative disease index scores (*right*) are shown. *p ≤ 0.05. Daily mean scores were analyzed by Mann-Whitney *U* and mean CDI by Student’s *t* test

Sex hormones have been shown to both positively and negatively regulate the immune system [24–26]. In order to evaluate the effect of female sex hormones on the treatment response in chronic EAE, WT and ovariectomized (OVX) C57BL/6 female mice were treated with 100 μg DRα1-mMOG-35-55. Unlike the lack of treatment effect in WT C57BL/6 female mice shown above, there was a significant treatment effect with this lower 100-μg dose in OVX females (Fig. 4, *p* < 0.05). To evaluate a specific role for estrogen in regulating the effective dose of RTL constructs, estrogen receptor (ER)α and ERβ knockout (ERKO and BERKO, respectively) mice were treated on day 20 post-immunization with 100 μg DRα1-mMOG-35-55, with boosting on days 35 and 49 p.i. as before. As shown in Fig. 5, DRα1-mMOG-35-55 treatment significantly reduced disease severity in ERKO female mice compared to vehicle-treated mice. Although EAE disease severity was lower in female BERKO vs. ERKO mice, only treatment of ERKO mice with the lower 100-μg dose of DRα1-mMOG-35-55 significantly reduced disease severity. Taken together, these data clearly demonstrate that estrogen signaling through ERα affects the potency of partial MHC class II constructs to treat chronic EAE.

## Discussion

Partial (p)MHC constructs were shown to be very effective in treating acute ongoing EAE [19, 27]. We demonstrated previously that this powerful treatment effect involves binding of the DRα1-mMOG-35-55 construct to CD74 on CD11b^+^ cells, resulting in blockade of MIF binding and downstream signaling and reduced CNS inflammation [14, 15, 17]. Moreover, DRα1-mMOG-35-55 treatment could enhance the frequencies of CD206^+^CD11b^+^ M2-like macrophages/microglia within the spinal cord with a striking reduction in expression of pro-inflammatory genes and enhancement of genes involved in neuro-survival and regeneration [17]. These anti-inflammatory and neuroprotective properties of pMHC constructs support their potential for treatment of chronic EAE. Herein, we demonstrate that two pMHC constructs, RTL342M and DRα1-mMOG-35-55, could successfully reduce the clinical severity and histological CNS damage in the chronic phase of EAE when given periodically during the transition from acute to chronic disease. Interestingly, the dose efficacy of this therapeutic effect was found to be sex dependent.

We demonstrated previously that pMHC were effective for treating acute EAE in different mouse strains when administered prior to or after disease onset [18, 19]. However, in order to treat chronic progressive MS, it is important to evaluate whether these constructs could be effective for treatment of chronic EAE, after the accumulation of axonal damage and ongoing demyelination. Indeed, our previous report showed that RTL401, an I-A^s^/PLP-139–151 construct, could reverse clinical disease and ongoing CNS damage in male SJL/J mice with relapsing-remitting EAE even when administered on day 20 post-disease induction [12]. In order to extend this initial observation to different mouse strains on the C57BL/6 background, we induced EAE in male and female DR*1501-Tg mice that express the major HLA-DR2 MS risk factor and started treatment with RTL342M on day 20 p.i., with boosting injections on days 35 and 49 p.i. As we observed previously in the SJL/J males, RTL342M treatment significantly reduced disease severity with significant correlations between reduced CDI vs. less damage and demyelination and increased axonal counts in male DR*1501-Tg mice compared to vehicle-treated mice. A similar treatment effect was observed using the DRα1-mMOG-35-55 construct to treat EAE in WT C57BL/6 males, with a 10-fold higher dose requirement for females. Thus, it was apparent that treatment with pMHCs in all three mouse strains resulted in reduced inflammation accompanied by varying degrees of myelin and axonal preservation and possibly some regeneration. Overall, the effects of treatment on myelin and axonal preservation seemed to be stronger in SJL/J mice than in C57BL/6-derived mice, due perhaps to a more severe chronic relapsing disease course (CDIs of ~150) than in chronic EAE in the C57BL/6 strains (CDIs of ~100).

Indeed, it is possible that the remyelination process is not affected directly by the pMHC constructs and that these constructs mainly reduce CNS inflammation, thus allowing remyelination. It was reported that the numbers of CD4^+^ T cells and F4/80^+^ cells in the CNS peak about at the same time as clinical disease scores (days 11–14 p.i.) followed by a continued decrease in cell numbers (days 26 and 40 p.i.) [28]. We also observed a significant reduction in CD4^+^ cell frequency in DR*1501-Tg mice in both RTL342M- and vehicle-treated mice on day 63 compared to day 20 p.i., which suggests that after disease peak, the pMHC treatment effect on CNS damage is not solely due to inhibiting peripheral immune cell migration into the CNS. This effect was more evident in treating C57BL/6 mice with DRα1-mMOG-35-55, where a significant effect on the daily EAE scores was achieved only after the boosting treatment, which started on day 35 p.i. These results suggest that perhaps higher or more frequent dosing would improve both the anti-inflammatory and neuroprotective activities of pMHC in future experiments.

Furthermore, we previously reported that pMHC enhanced the frequency of anti-inflammatory M2-like CD11b^+^ cells in the CNS during EAE [17]. Miron et al. demonstrated that M2-like microglia could promote oligodendrocyte progenitor cell (OPC) differentiation after lysolecithin-induced demyelination, even in the absence of infiltrating macrophages, suggesting that the M1/M2-like switch to an anti-inflammatory milieu is crucial for remyelination and might not depend solely on inhibition of peripheral inflammation [29]. Taken together with our previous report demonstrating that RTL constructs could be detected in the CNS parenchyma of successfully treated EAE mice [30], these data suggest that DRα1-mMOG-35-55 could enter the CNS and bind to CD11b^+^ cells and affect their polarization state.

Interestingly, treatment of chronic EAE in DR*1501-Tg mice with 100 μg RTL342M per injection or of C57BL/6 mice with 100 μg DRα1-mMOG-35-55 per injection revealed a sex-dependent effect. This finding would not have been observed in our previous report due to the exclusive use of SJL/J males in that study. It is important to note that such differences between males and females in the ability of pMHC to treat EAE were not observed when mice were treated at disease onset at a clinical score of ≥2. In the current study, EAE in female C57BL/6 mice could be treated only when we administered a 10× dose of 1 mg DRα1-mMOG-35-55 per injection. It is noteworthy that a similar sex-dependent dose effect was observed when DRα1-mMOG-35-55 was used to treat experimental stroke in DR*1502-Tg mice. Importantly, effective stroke treatment in female mice was also observed with the 1-mg dose vs. the 100-μg dose used in males [23]. As in other autoimmune diseases such as relapsing-remitting MS, there is a strong gender bias of disease being two to three times more common in women than in men. This ratio decreases with older age and there is an equal sex ratio among progressive patients [31–34]. However, in EAE, this difference is dependent on the mouse strain and on the neuroantigen that is used for disease induction. We did not observe any difference in disease course between female and male DR*1501-Tg or C57BL/6 mice [35].

To further address the underlying cause of this sex-specific reduction of potency of DRa1-mMOG-35-55 treatment of chronic EAE, we evaluated the potential requirement for female sex hormones. Our results indicate that ovariectomy of female mice could restore treatment efficacy in chronic EAE at the lower 100-μg dose of drug. We do not expect that a sham operation would affect these results. The successful low-dose treatment of chronic EAE in ERKO but not BERKO female mice demonstrated that presence of ERα is directly linked to the sex-specific reduction in DRa1-mMOG-35-55 potency. It is widely accepted that sex hormones are involved in regulating the immune response, although the relevant mechanisms are not fully understood. We and others demonstrated that low dose as well as pregnancy levels of estrogen could induce protection against EAE [26, 36]. It was also reported that estrogen is involved in pro-inflammatory responses [25]. It is possible that during chronic EAE, estrogen signaling through ERα upregulates CD74 and/or MIF, which are involved in disease progression. Thus, higher concentrations of partial MHC class II might be required for effective treatment. It was reported that MIF and CD74 are upregulated in subjects with breast cancer (BC) [37, 38]. In addition, Wu et al. compared two BC cell lines, MCF-7 which is ER^+^ and MDA-MB-231 which is ER^−^, and showed that higher CD74 mRNA expression was associated with ER^+^ cells compared to ER^−^ cells [39]. Taken together with our results evaluating pMHC ability to downregulate CD74 in PBMC from human subjects, these results indicate that pMHC constructs were significantly more effective in downregulating CD74 on PBMC from male subjects compared to female subjects (manuscript in preparation). These findings suggest that female sex hormones might affect the partial MHC class II treatment efficacy by altering the expression levels of CD74 and MIF.

## Conclusions

In summary, we showed that administration of pMHC constructs during chronic EAE not only reduced disease severity but also markedly reduced ongoing demyelination and axonal damage in the CNS. We further demonstrated that the effective dose of these constructs is sex dependent and might be regulated by estrogen signaling through ERα. Our findings will further assist future clinical applications of the partial MHC class II constructs by suggesting the need to correctly dose females with progressive MS.

## Additional file

Additional file 1: Figure S1. EAE disease course in female and male (A) DR*1501-Tg and (B) C57BL/6 mice, immunized with mMOG-35-55 peptide. **Figure S2.** Days 20 and 63 p.i. spinal cord lumbar sections from EAE female DR*1501-Tg mice were stained with luxol fast blue (LFB) and analyzed for demyelination, toluidine blue for spinal cord damage, and CD4^+^ cell frequency in the spinal cords of RTL342M- vs. vehicle-treated mice. (PDF 231 kb)

## Abbreviations

BERKO: Estrogen receptor beta knockout
CDI: Cumulative disease index
CNS: Central nervous system
EAE: Experimental autoimmune encephalomyelitis
ERKO: Estrogen receptor alpha knockout
ERα: Estrogen receptor α
ERβ: Estrogen receptor β
LFB-PAS: Luxol fast blue-periodic acid-Schiff
M1: Classically activated macrophages
M2: Alternatively activated macrophages
MIF: Macrophage migration inhibitory factor
MS: Multiple sclerosis
OVX: Ovariectomized
p.i.: Post-immunization
pMHC: Partial major histocompatibility complex
RR: Relapsing-remitting
RTL: Recombinant T cell ligand
SP: Secondary progressive
