# Supplementary material for: Sex-dependent treatment of chronic EAE with partial MHC class II constructs
Source: J Neuroinflammation. 2017 May 6;14:100. doi: 10.1186/s12974-017-0873-y (PMC5420407; doi:10.1186/s12974-017-0873-y)
Supplement: Additional file 1: Figure S1. — EAE disease course in female and male (A) DR*1501-Tg and (B) C57BL/6 mice, immunized with mMOG-35-55 peptide. Figure S2. Days 20 and 63 p.i. spinal cord lumbar sections from EAE female DR*1501-Tg mice were stained with luxol fast blue (LFB) and analyzed for demyelination, toluidine blue for spinal cord damage, and CD4+ cell frequency in the spinal cords of RTL342M- vs. vehicle-treated mice. (PDF 231 kb) [file 12974_2017_873_MOESM1_ESM.pdf]

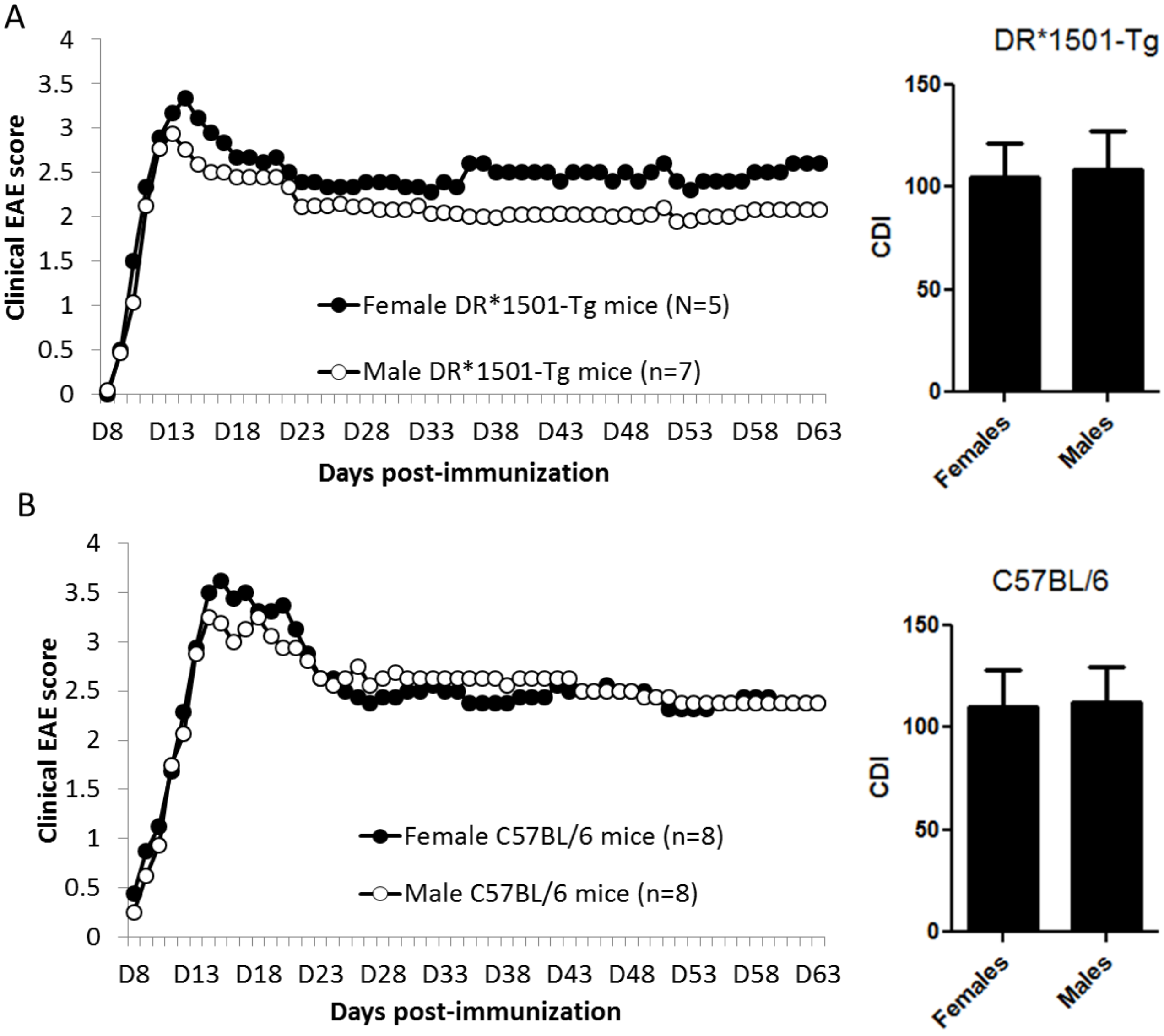

Figure S1. EAE disease course in female and male (A)DR*1501-Tg and (B) C57BL/6 mice, immunized with mMOG-35-55 peptide.

Female DR*1501-Tg mice

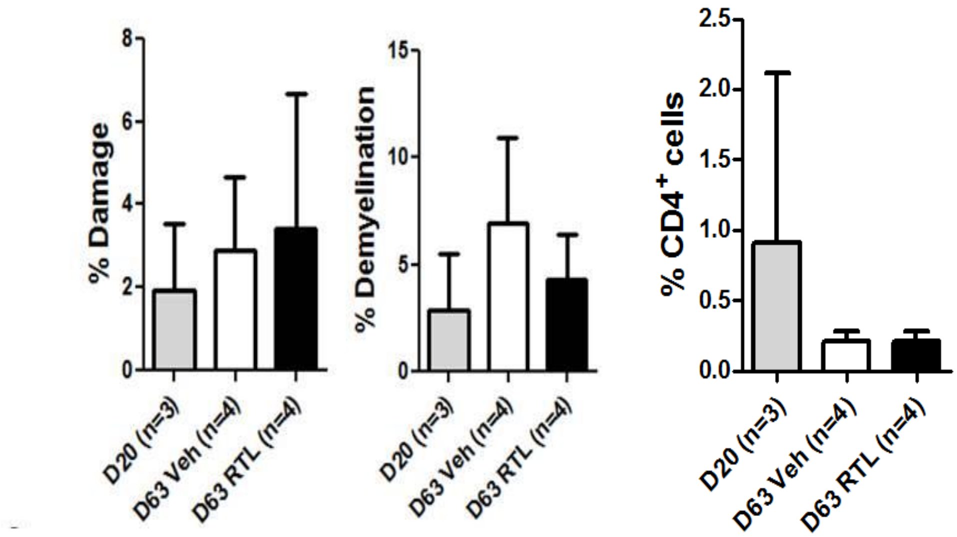

Figure S2. Days 20 and 63 p.i. spinal cord lumbar sections from EAE female DR*1501-Tg mice were stained with luxol fast blue (LFB) and analyzed for demyelination, Toluidine blue for spinal cord damage and $CD4^{+}$ cells frequency in spinal cords of RTL342M vs. vehicle treated mice.
